# Serum Proteomic Analysis Reveals High Frequency of Haptoglobin Deficiency and Elevated Thyroxine Level in Heroin Addicts

**DOI:** 10.1371/journal.pone.0095345

**Published:** 2014-04-17

**Authors:** Bing-Ying Zhou, Shi-Yan Yan, Wan-Lu Shi, Zhi Qu, Xin Zhao, Zhi-Min Liu, Xiao-Ping Pu

**Affiliations:** 1 National Key Research Laboratory of Natural and Biomimetic Drugs, Peking University, Beijing, People’s Republic of China; 2 Department of Molecular and Cellular Pharmacology, School of Pharmaceutical Sciences, Peking University, Beijing, People’s Republic of China; 3 National Institute of Drug Dependence, Peking University, Beijing, People’s Republic of China; Hokkaido University, Japan

## Abstract

Heroin addiction is a chronic, complex disease, often accompanied by other concomitant disorders, which may encumber effective prevention and treatment. To explore the differences in expression profiles of serum proteins in control and heroin addicts, we used two-dimensional electrophoresis coupled to MALDI-TOF/TOF, and identified 4 proteins of interest. Following validation of the increase in serum transthyretin, we assessed serum levels of thyroid stimulating hormone (TSH), triiodothyronine (T3), and thyroxine (T4), and observed a robust increase in T4 in heroin addicts compared to controls. In addition, we performed haptoglobin (Hp) phenotyping, and showed that the frequency of Hp0 (serum devoid of haptoglobin) was significantly higher in heroin addicts. Altogether, these findings indicated that: (1) thyroid hormone imbalance is present in heroin addicts; (2) anhaptoglobinemia (Hp0) might a risk factor or a deleterious effect of heroin abuse.

## Introduction

Heroin is the most prevalent drug of abuse in China, according to *World Drug Report 2013* (United Nations Office on Drugs and Crime). Even worse, the number of registered heroin consumers keeps rising rapidly, reaching 1.272 million by the end of 2012. As an illegal and highly addictive drug, heroin causes serious medical, economic and social problems, threatening the society [1]. Despite great effort in Methadone Maintenance Treatment (MMT) programs to alleviate heroin dependence, effective prevention and treatment still remain obscure, fueling the need for more research into the causes and consequences of the heroin abuse.

Interestingly, genetic predisposition is a major contributing factor of the development of addiction. Epidemiological studies in men demonstrated that approximately 40–60% of developing an addiction to heroin is genetically determined [2]. At the genome level, a number of genes have been identified accounting for either risk of or protection from developing addiction toward heroin [3]–[9]. However, these genetic underpinnings have not been exploited for clinical benefit.

The deleterious consequences of heroin dependence are largely known as cognitive deficits and lethality to the individual, and impairment of economy and public safety to the society. Studies have shown that parts of the central nervous system, including dynorphin-kappa opioid system [10] and the endocannabinoid system [11], are heavily involved in drug addiction, which provide intriguing targets for developing therapies. A comprehensive understanding of the pathophysiological changes in drug users may lead to potential treatments improving their quality of life.

The emergence of the terms “morphinome” and “cocainomics” underscored the importance of proteomic research in opiate addiction [12], [13]. Extensive proteomic studies have been conducted on opiates. However, efforts in revealing the proteomes in heroin addiction remain few [14]–[18]. To the best of our knowledge, no proteomic studies using human samples have been reported. Studies on human subjects pose an enormous challenge for sampling. Brain tissues are the best samples for laboratory research on central nervous system disorders such as addiction [19], [20]. However, postmortem tissues of heroin addicts are not easily obtainable. A favorable surrogate is the cerebrospinal fluid, which is usually obtained via lumbar, cisternal or ventricular puncture [21]. Blood, by contrast, can be collected much more easily and at much lower risks. Importantly, it contains nearly the entire proteome of the human body [22]. Therefore, our study used human serum.

To uncover proteins potentially involved in heroin addiction, we utilized a two-dimensional electrophoresis (2-DE)-based proteomics approach to examine differential protein expression in the serum of heroin addicts and healthy controls. Following the identification differentially expressed proteins, we validated the increase in serum transthyretin levels in heroin-addicted patients, and further found that thyroid hormone thyroxine (T4) was elevated. In addition, we performed haptoglobin phenotyping, and showed, for the first time, that patient serum devoid of haptoglobin (anhaptoglobinemia) positively correlated with heroin dependence. These findings indicate that thyroid hormone balance is disrupted in heroin addicts, and that anhaptoglobinemia might be a genetic risk factor or a pathological consequence of the disease, which could provide clues for the prevention or treatment of heroin addiction.

## Materials and Methods

### 1. Subjects

This study was approved by the Human Subjects Division at Peking University Health Science Center. All subjects were of Han Chinese descent. To preclude the effect of gender on the serum proteome, only male individuals were included in this study [23]. All surveys were conducted between November 2009 and January 2010. Heroin-addicted patients were consecutively recruited through several different clinics/hospitals. They were Beijing Community Methadone Maintenance Treatment (MMT) First Clinic, No. 109 Hospital of Shanxi, Xi’an Beilin District MMT Clinic, Xi’an Baqiao Chinese Medicine Hospital MMT Clinic, and Xi’an Mental Health Center MMT First and Second Clinic. All patients were interviewed by two independent clinicians at each institution. The heroin addiction group comprised 110 individuals (mean age of 33.6±7.5 years, male, Table 1). The subjects were selected based on the Diagnostic and Statistical Manual of Mental Disorders, 4th Edition (DSM-IV.) the Addiction Severity Index (ASI). To be included in the study, subjects had to meet the following criteria:(1) men aged 18 or above; (2) heroin is a predominant drug of abuse; (3) currently on methadone maintenance treatment; (4) adhered sufficiently to the study protocol. The exclusion criteria were as follows: (1) concurrent use of multiple drugs, predominant drug indefinable; (2) severe psychological disorders; (3) severe physical illnesses (e.g., kidney failure, hepatic failure); (4) mental retardation, incapable of understanding questionnaire. The first group of healthy controls were volunteers recruited through Peking University Third Hospital. All controls were college student volunteers (male, aged 18–22) from Beihang University (previously “Beijing University of Aeronautics and Astronautics”), who underwent health examinations at Peking University Third Hospital (Table 1). The extended cohort of healthy volunteers were recruited from the community, which met the following criteria: (1) healthy male; (2) 18 years or above; (3) no history of illicit drug abuse. An extended cohort of 72 male heroin addicts (35.51±8.45 years) and healthy controls (32.79±8.63 years) were recruited for thyroid hormone measurements. This study complied with the guidelines of each institution, and was approved by each Ethics Committee. All participants recruited in this study provided written informed consent.

**Table 1:** Demographic information of subjects.

	Controls	Heroin addicts
Number	110	110
Male : Female	110∶ 0	110∶ 0
Race	Han Chinese	Han Chinese
Age	18–22	33.6±7.5 (mean ± SD)

Detailed information about the number (n), sex (male:female) and age of controls and heroin addicts are shown in this table.

### 2. Serum Sample Preparation

Approximately 5 ml of blood was drawn by venipuncture, and collected into unheparinized tubes. According to the Human Proteome Organization (HUPO) reference sample collection protocol [24], all blood samples were allowed to clot at room temperature for 30 min, centrifuged at 1,300×*g* (4°C) for 10 min. Serum used for proteomic experiments were additionally re-centrifuged at 1,300×*g* (4°C) for 10 min to remove all residual cell debris. The resulting supernatants were aliquoted and frozen at −80°C until use.

### 3. Two-dimensional Gel Electrophoresis (2-DE)

Twenty samples were randomly selected from each group for 2-DE. Each sample corresponded to one gel. Protein extracts from control and patient serum were processed in parallel to ensure maximal comparability. Albumin was removed from serum samples as described previously [25]. In short, serum was precipitated by addition of ice-cold acetone containing 10% (w/v) trichloroacetic acid, and subsequently washed with acetone four times. Total protein concentration was determined using a Bradford Protein Assay Reagent Kit (Thermo Scientific). 2-DE was performed as reported previously [26]. A protein load of 1.3 mg protein was applied to 17 cm IPG Strips (pH 4–7, GE Healthcare). Focusing was performed according to the protocol provided by Bio-Rad Laboratories. The second-dimensional separation was performed on 12.5% SDS-polyacrylamide gels. After fixation, gels were stained using colloidal Coomassie Blue (CCB) with slight modifications [27]. Gels were scanned, and analysis for differences in protein patterns was performed using PDQuest (Version 7.1, Bio-Rad).

### 4. Mass Spectrometry Analysis

Protein spots of interest were excised from 2-DE gels, washed, dried, and enzymatically digested with trypsin. The extracted peptide solution was subjected to the Applied Biosystems 4700 Proteomics Analyzer MALDI-TOF/TOF for MS/MS analysis. The peptide masses were searched against the National Center for Biotechnology Information (NBCI) database using Mascot Search from Matrix Science. A criterion for significance was defined using a probability-based molecular weight search (MOWSE) score. Individual scores greater than 56 indicated identity or extensive homology (*P*<0.05).

### 5. Western Blot Analysis

Patient and control serum sample were separated by SDS-PAGE, and transferred onto polyvinylidene difluoride (PVDF) membranes (Millipore, USA). TTR (or “prealbumin”) was detected using a polyclonal rabbit anti-prealbumin antibody (Santa Cruz), followed by incubation with a secondary HRP-conjugated goat anti-rabbit antibody (Proteintech Group). To ensure equal protein loading, membranes were also probed with a polyclonal rabbit anti-GAPDH antibody (Santa Cruz, USA) [28]. Blots were developed using enhanced chemiluminescence (ECL kit; Amersham). Relative band intensities were determined by Quantity One (Bio-Rad). Western blot shown is representative of seven independent experiments.

### 6. Radioimmunoassay

For each participant, serum levels of thyroid stimulating hormone (TSH), triiodothyronine (T3) and thyroxine (T4) were determined by radioimmunoassay. The assay was performed according to manufacturer’s protocol (Xinwan Biology and Science, Tianjin, China), with intra-assay coefficients of variation less than 10.0% and inter-assay coefficients of variation less than 15.0%.

### 7. Haptoglobin Phenotyping

All serum samples from each group were subjected to haptoglobin (Hp) phenotyping. A 10% hemoglobin (Hb) solution was prepared as previously reported [29]. In brief, heparinized blood was centrifuged at 3,000 rpm (4°C) for 10 min, and the supernatant was discarded. The pelleted blood cells were washed five times in phosphate buffered saline (PBS), and lysed in 9 ml of sterile water per 1 ml of pelleted cell volume. The cell lysate was centrifuged at 10,000×*g* (4°C) for 40 min, and the resulting supernatant was aliquoted and stored at −80°C until use. The Hp-Hb complex solution was prepared by adding 2 µl of 10% Hb solution to 10 µl of serum, and mixing for 5 min at room temperature [30]. Sample buffer (25 mM Tris and 192 mM glycine) was added to each sample prior to electrophoresis. Samples were resolved by 7.5% PAGE at 300 V for 3 h, after which Hp-Hb complexes were visualized by immersing the gel in a freshly prepared staining solution containing 10 ml of 0.2% (w/v) 3,3′,5,5′-tetramethylbenzidine in methanol, 1 ml DMSO, 20 ml of 5% (v/v) glacial acetic acid, 2 ml of 1% (w/v) K_3_[Fe(CN)_6_], and 300 µl of 30% H_2_O_2_.

### 8. Statistical Analysis

Statistical analysis was performed with the SPSS Statistics software (version 17.0). Unless otherwise indicated, the two-tailed Student’s *t*-test was performed. In Hp phenotyping, the chi-square (χ^2^) goodness-of-fit test (one sample test) was used for each single Hp phenotype distribution, and the Fischer’s exact test was used to compare Hp phenotype frequencies between the two groups. A *P* value of less than 0.05 was considered statistically significant.

## Results

### 1. 2-DE of Control and Heroin Addict Serum

**Figure 1:**
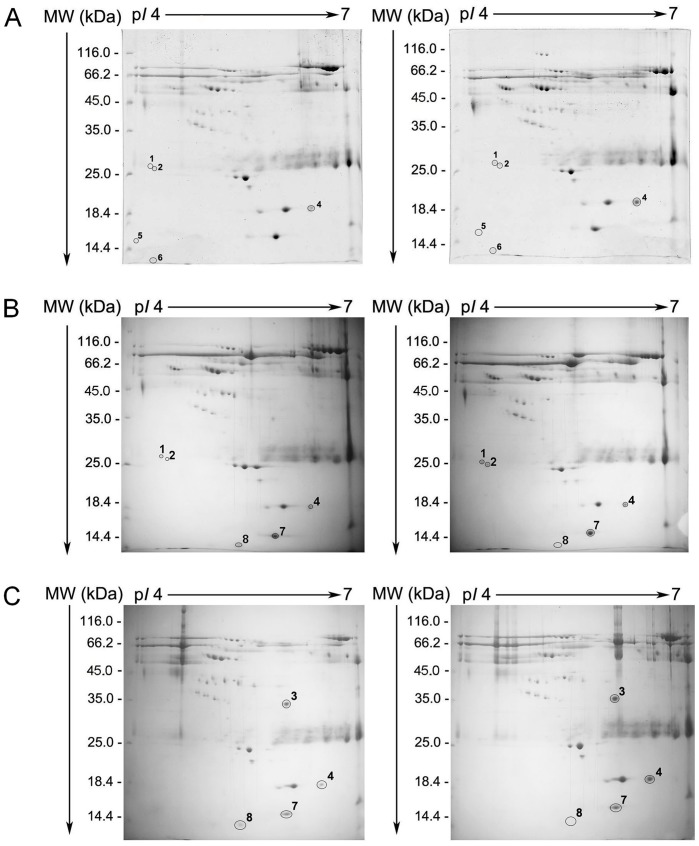
Representative raw 2-DE gel images of the serum proteome. Two-dimensional separation of the serum proteomes of controls (left) and heroin addicts (right) were visualized by colloidal Coomassie brilliant blue. Protein samples were separated using IPG gel (pH 4–7, 17 cm) in the first phase, followed by 12.5% SDS-PAGE in the second phase; 1.3 mg of proteins were loaded onto each gel. (A)–(C): protein spots marked 1–8 exhibited differential expression.

**Figure 2:**
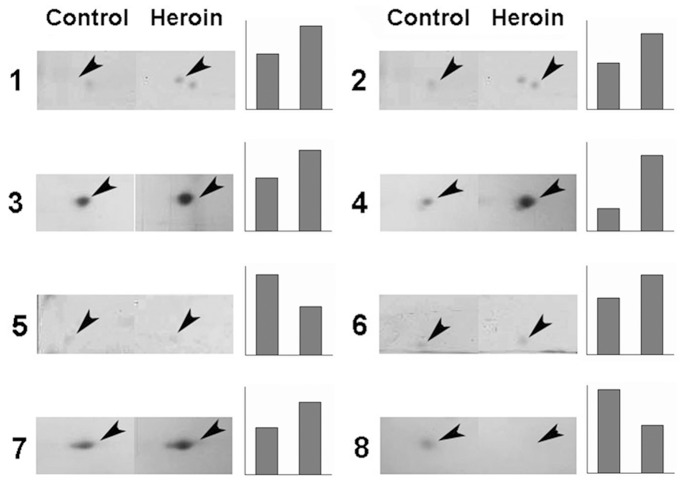
Relative abundance of eight differentially expressed proteins. Enlarged regions of the corresponding gels are shown for all 8 differentially expressed proteins. The proteins (No. 1–8) indicated by the arrows exhibited robust differences in their expression between the control group and heroin addict group. The graphs show the relative abundance of the proteins indicated across the two gels. The scales of the graphs are in arbitrary units.

Serum proteins extracted from controls and heroin addicts were analyzed in parallel using 2-DE followed by colloidal Coomassie Blue staining. Due to the variations among individuals, 2-DE maps do not consistently exhibit all proteins. Therefore, we selected 3 pairs of 2-DE images as references for spot picking (Figure 1). The spot patterns are summarized in Figure 1. All gels were analyzed using PDQuest (Bio-Rad). A total of 113 spots from the heroin addict group could be matched to the control group with a match rate of 49%. Of the protein spots with different staining intensities between control and heroin addicts, eight spots were selected for further analysis. The relative abundance of spots 1–8 between the two groups was analyzed by PDQuest (Table 2). Spots with *P*<0.05 was considered significantly differentially expressed between heroin addicts and healthy controls, which included spot numbers 2, 3, 4, 7 and 8 (Table 2). Enlarged regions of the respective gels are shown in Figure 2 (Figure 2).

**Table 2:** Quantitative changes in differentially expressed proteins in controls versus heroin addicts.

	Control		Heroin		
Spot no.	Spot density	CV (%)	Spot density	CV (%)	*P* value (*n* = 3)
1	473.67	26.52	642.19	17.40	0.158
2	353.96	1.78	755.49	20.09	0.010
3	1161.86	55.89	2501.54	19.08	0.045
4	576.89	28.87	2257.15	30.68	0.015
5	1245.40	0.02	704.03	34.50	0.088
6	759.74	22.79	1146.57	41.58	0.257
7	1525.20	29.27	2177.21	26.054	0.042
8	1829.03	29.22	362.57	9.46	0.009

Spot numbers correspond to the same numbers shown in Figure 1. Spot density is the average OD of the same spot from three independent samples belonging to the same experimental group. CV shows spot OD variation across these three samples.

### 2. Identification of Differentially Expressed Proteins by Mass Spectrometry

**Figure 3:**
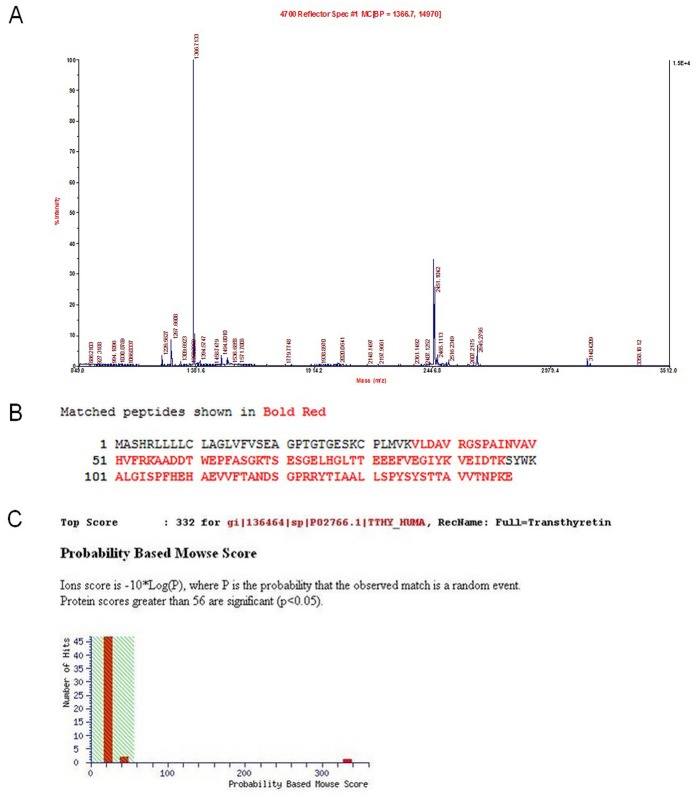
Representative mass spectrometry output and identification of spot No. **(**A) The peptide mass fingerprint of spot 3 from human serum analyzed with MALDI-MS. (B) Identification of spot 3 as transthyretin with 73% amino acid sequence coverage by matched peptides using MASCOT software. (C) MASCOT search of the protein in the NCBI database.

The eight spots were excised from the gels, digested with trypsin and analyzed with MALDI-MS. Peptide masses were used to query the NCBI database, and the eight spots were all significant with MOWSE scores greater than 56. The results, including protein names, accession numbers, the number of matching peptides, the percentage of sequence coverage, the theoretical MW and p*I*, and spot intensity variations are included in Table 3. There were 6 up-regulated and 2 down-regulated protein spots. The 6 protein spots with an increase upon heroin dependence were identified as immunoglobulin J chain (spots 1 and 2), transthyretin (spot 3 and 7), haptoglobin (spot 4), and vitronectin (spot 6). The 2 spots exhibiting a decrease in expression were vitronectin (spot 5) and haptoglobin (spot 8). Figure 3 reports the peptide mass fingerprint and identification of transthyretin (spot 3) to provide an example of the peptide mass fingerprints of the eight protein spots (Figure 3). As can be seen from Tables 2 and 3, of the four proteins (peptides) identified, only transthyretin (TTR) showed a consistent up-regulation at both spots (No. 3 and 7), both of which reached statistical significance (*P* = 0.045 and *P* = 0.042, respectively).

**Table 3:** Identification of proteins differentially expressed between control and heroin addict sera.

Spot No.	Protein identity	Accession no.	Sequence coverage (%)	MW (kDa)	Ratio Heroin/Control	Score	Description
1	Immunoglobulin J chain	gi|261260100	35	18.543	1.36	163	IgM and IgA polymerization
2	Immunoglobulin J chain	gi|261260100	49	18.543	2.13	191	IgM and IgA polymerization
3	Transthyretin	gi|136464	73	15.991	2.15	332	Transports thyroxine and retinol
4	Haptoglobin	gi|123508	20	45.861	3.91	132	Binds to free hemoglobin
5	Vitronectin	gi|139653	5	55.069	−1.77	157	Serum spreading factor
6	Vitronectin	gi|139653	5	55.069	1.51	91	Serum spreading factor
7	Transthyretin	gi|136464	73	15.991	1.43	779	Transports thyroxine and retinol
8	Haptoglobin	gi|123508	16	45.861	−5.04	211	Binds to free hemoglobin

Spot numbers correspond to the same numbers shown in Figure 1. MW is the theoretical molecular mass calculated from the amino acid sequence. p*I* is the theoretical p*I* calculated from the amino acid sequence of the predicted mature protein. Ratio Heroin/Control represents the ratio of spot intensity of the heroin addict to that of the same spot in the control group.

### 3. Validation of Serum Transthyretin Expression

**Figure 4:**
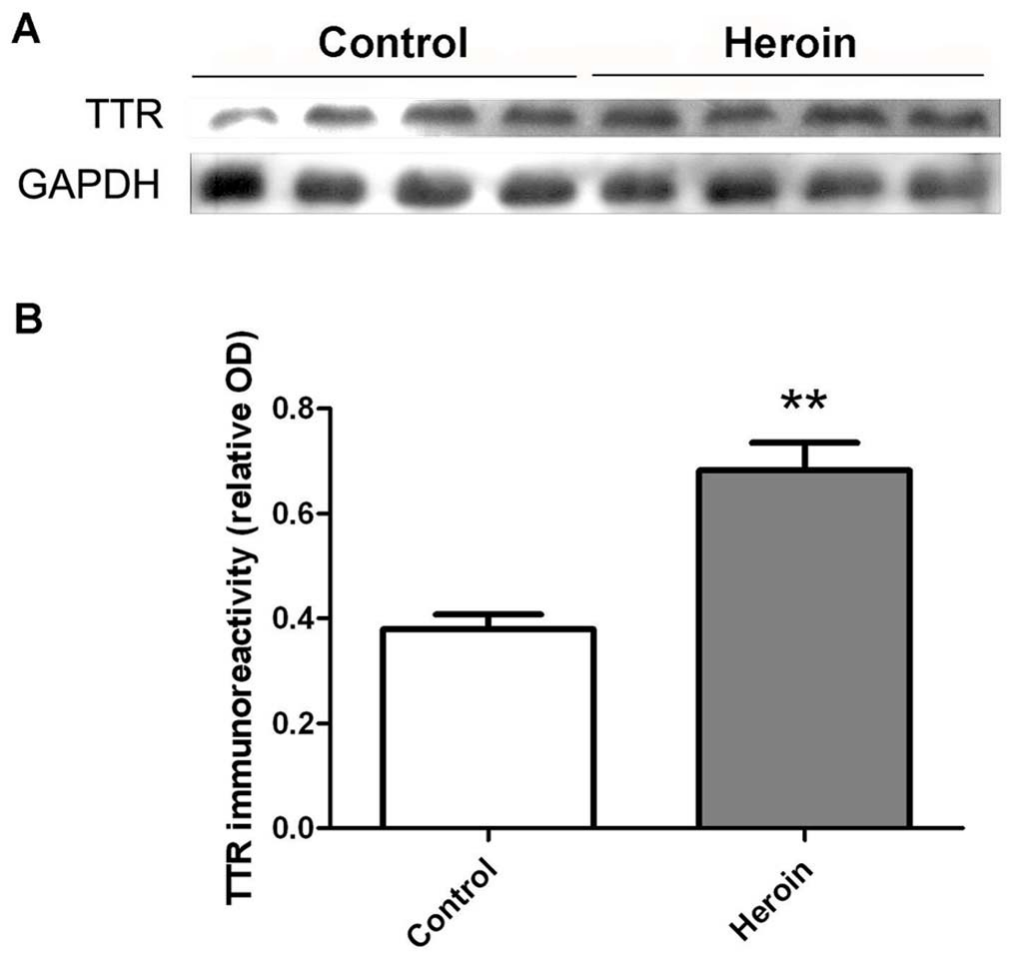
Western blot analysis of TTR in the serum of control and heroin addicts. (A) Representative blots showing relative amounts of TH and GAPDH in control and heroin addicts. (B) Statistical analysis of the relative optical density (OD) of the detected bands. Results are shown as means ± S.E.M. from seven independent experiments. **P<0.01, v.s. control.

To further confirm the protein abundance changes observed in 2-DE, western blotting for serum TTR levels were performed. As shown in Figure 4, serum TTR level was increased in heroin addicts (*P*<0.01), which is consistent with the results from 2-DE (Figure 4). Together, these data suggest that TTR is altered in heroin-addicted patients.

### 4. Levels of Thyroid Hormones in Controls and Addicts

Transthyretin (TTR) is one of the three thyroxine (T4)-binding proteins in human serum [31]. The increase in serum TTR in heroin addict indicated that the thyroid hormone T4 might be deregulated in addiction. To this end, we examined serum levels of thyroid stimulating hormone (TSH), triiodothyronine (T3) and thyroxine (T4) in controls and heroin addicts, and found a marked increase in T4 level in addicts (Table 4), whereas T3 and TSH levels were not significantly different between cases and controls.

**Table 4:** Comparison of TSH, T3 and T4 concentrations (nmol/L) in the serum of healthy controls versus heroin addicts.

Thyroid hormone	Control	Heroin	*P* value
TSH	3.69±1.14	3.61±1.63	0.713
T3	1.72±0.58	1.53±0.71	0.093
T4	91.58±38.04	114.02±25.24	<0.001***

***P<0.001, *vs*. control.

### 5. The Distribution of Haptoglobin Phenotypes in Control and Heroin Group

As shown in Tables 2 and 3, spots 4 and 8, both identified as haptoglobin, showed alterations in different directions. According to the literature, spot 4 represents the α_2_ chain of the protein, whereas spot 8 is the α_1_ chain [32]. This led us to the hypothesis that Hp abundance may not necessarily be related to heroin addiction, instead, Hp polymorphism, dictated by different combinations of α chain isoforms, may be a potential factor influencing susceptibility to heroin addiction. Therefore, we performed Hp phenotyping to determine the relationship between Hp phenotype and heroin addiction.

**Figure 5:**
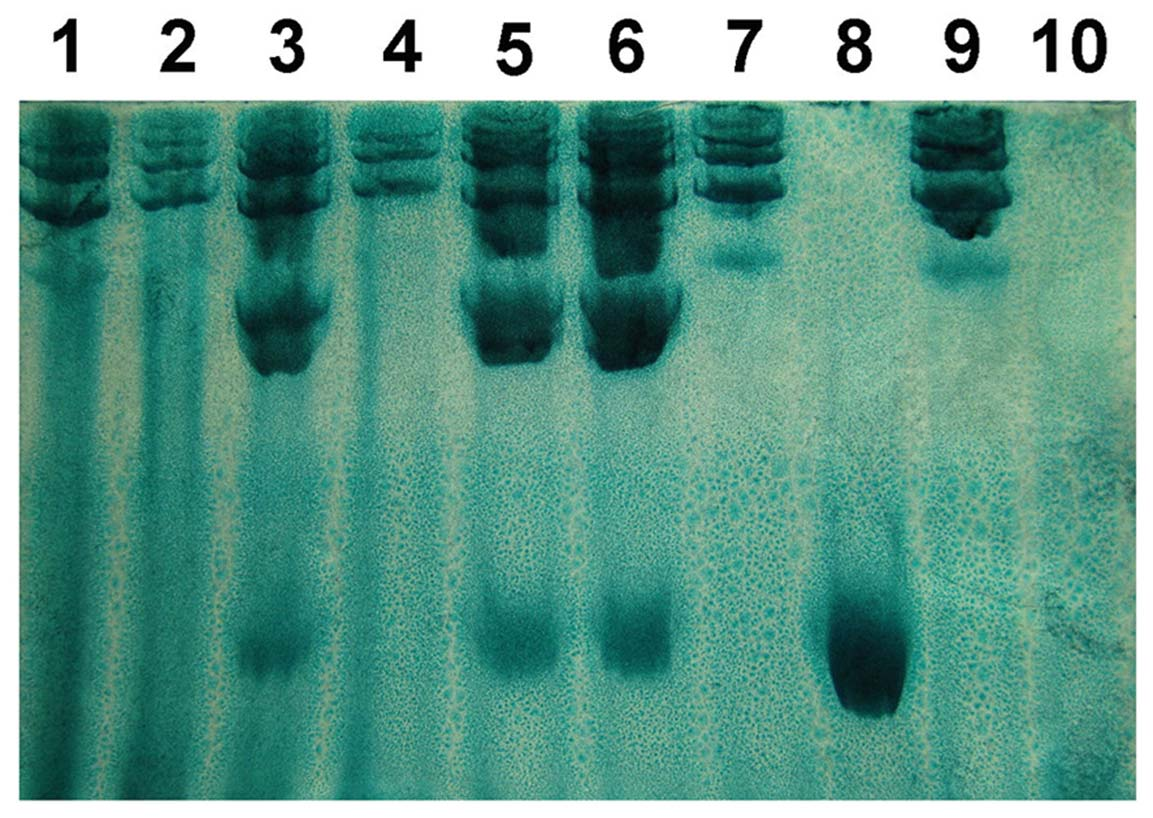
Representative patterns of Hp phenotypes after polyacrylamide gel electrophoresis of hemoglobin-enriched serum. Bands correspond to Hp-hemoglobin complexes. Hp1-1 shows a single migrating band. Hp2-2 has a series of more slowly migrating bands. Hp2-1 displays another series of more slowly migrating bands and a weak band that migrates similar to the Hp1-1 band. Hp1-1: lane 8; Hp2-1: lanes 3, 5 and 6; Hp2-2: lanes 1, 2, 4, 7 and 9; Hp0: lane 10.

The three major Hp phenotypes (Hp1-1, Hp2-1, and Hp2-2) and serum not expressing Hp (Hp0) were easily distinguished by characteristic patterns of bands representing the Hp-Hb complexes (Figure 5). The frequencies of Hp1-1, Hp2-1, Hp2-2 and Hp0 are demonstrated in Table 5. Hp2-1 displayed an increase in frequency in the heroin addiction group, whereas the frequency of Hp2-2 decreased. Hp0 was present only in the heroin addiction group.

**Table 5:** Haptoglobin phenotype frequencies and statistical analyses in control and heroin addicts.

	Hp phenotype counts (frequency)						
Group	Hp1-1	Hp2-1	Hp2-2	Hp0	Total	?^2^ b	*P* b
Control	12 (0.109)	37 (0.336)	61 (0.554)	0 (0.000)	110		
Heroin	11 (0.100)	46 (0.418)	48 (0.436)	5 (0.045)	110		
**Total**	23	83	109	5	220	7.448	0.0514
**χ^2^** a	0.043	0.976	1.550	5.000			
***P*** a	0.835	0.323	0.213	≈0.025*^c^			

a Chi-square goodness-of-fit test.

b Fisher’s exact test.

c Exact *P* value not applicable (if χ^2^ = 5.02 and df = 1, then *P* = 0.025).

Next, we assessed the distribution of each Hp phenotype using the chi-square goodness-of-fit test (one sample test). Hp1-1, Hp2-1, Hp2-2 frequencies did not show marked differences between the two groups, whereas was found to be significantly differently distributed (P≈0.025). Fisher’s exact test was used to compare the distribution of Hp phenotypes in cases and controls. This test was preferred over a parametric test because there were instances of small cell counts in the tables. No substantial differences were revealed. However, a *P* value of 0.0514, which is close to the critical value of 0.05, suggests that there might be potential differences in the distribution of Hp phenotypes if further increasing sample size. These results indicate a possible correlation between the lack of Hp and heroin addiction.

## Discussion

Heroin addiction is a major public health problem worldwide. It is widely believed that the mesolimbic dopamine pathway plays a pivotal role in drug reward and addiction, and that dopamine release is central to the reinforcing effects of drugs of abuse [33]. Imaging studies have described blunted presynaptic dopamine release and low D2-receptor binding as an important part of the pathophysiological features characterizing chronic heroin use [34]. Despite extensive effort in uncovering the neuropathophysiology underlying heroin addiction, no cure exists for the disease. The most widely implemented treatment mode is maintenance treatment, which is a process of administering substitutes such as methadone, buprenorphine, or sometimes heroin itself, to gradually help patients return to productive lives [35], [36]. However, maintenance treatments are controversial because in essence, patients remain physically dependent on opiates for up to their entire lives. Therefore, better therapies are sought after. Unveiling biochemical differences between heroin-addicted patients and healthy controls may lead to the identification of risk factors, “druggable” targets, or sophisticated pathways and networks orchestrating the disease, which lays the foundations for effective prevention or treatment for heroin addiction.

Proteomic techniques are broadly applied in human disease for the discovery of differentially expressed proteins. Here, we used 2-DE and tandem MS, and identified 4 proteins (peptides) of interest. They were immunoglobulin J chain, transthyretin, haptoglobin, and vitronectin. As opposed to gel-free proteomics, this detection method sets the detection threshold at a very high level, which can be easily converted and applied in a clinical setting.

Transthyretin (TTR) is a 55-kDa homotetramer with a dimer of dimers configuration, and sometimes exists in the form of monomers as well [37], [38]. Accordingly, spots 3 and 7 (respective molecular weight estimates of 30 kDa and 15 kDa), are the TTR dimer and monomer, respectively. Physiologically, TTR is a serum and cerebrospinal fluid carrier of the thyroid hormone thyroxine (T4) and retinol [31]. In human serum, approximately 15% of T4 is transported via TTR. Several studies have focused on the dysregulation of thyroid hormones in heroin addiction. Despite the prevailing view that thyroid function is disrupted in heroin addicted patients, major controversies exist. Some early studies showed that both T3 and T3 levels were increased in heroin addicts [39]–[41]. Rasheed et al observed a modest gain in T3, with no alterations in either TSH or T4 [42]. In yet another study, no differences were found between controls and addicts with regard to basal levels of TSH, T3 and T4 [43]. Our results, by contrast, demonstrated a significant increase in T4, but not T3 or TSH, in the serum of male heroin addicts compared with healthy controls, which has not been previously reported. Additionally, the carrier protein of T4, TTR, was upregulated at the protein level. Although not consistent between, the imbalance observed by all groups indicates abnormal thyroid function in heroin dependence.

Haptoglobin (Hp) is an acute phase protein that binds free hemoglobin and eliminates it from the circulation to prevent kidney injury and iron loss [44]. Hp polymorphism is the result of two co-dominant allele variants (Hp1 and Hp2) on chromosome 16q22, encoding the Hp α chains [45]. The Hp molecule contains 2 types of polypeptide chains: α (α_1_, 8.9 kDa; α_2_, 16 kDa) and β (40 kDa). The β chains are identical in all Hp phenotypes, and variations are solely caused by the presence of different α chains. Three major Hp phenotypes are known to exist: Hp1-1, Hp2-1 and Hp2-2. Some individuals do not express Hp (anhaptoglobinemia), which is denoted by Hp0. In this study, we demonstrated that the frequency of Hp0 phenotype was significantly higher in heroin addicts than in healthy controls, which is the first report on anhaptoglobinemia in the context of heroin addiction. However, whether it is a risk factor conferring vulnerability to addiction, or a consequence of heroin use [46], is still unclear. Further, the presence of both congenital and acquired Hp0 adds another layer of complexity to the elucidation of the causal relationship [47].

The other two proteins (peptides) identified were immunoglobulin J (IGJ) chain and vitronectin. IGJ participates in the polymerization of the antibodies Immunoglobulin A (IgA) and Immunoglobulin M (IgM). Serum IgA is a monomer, and does not require the presence of IGJ chains. Therefore, the identified IGJ chains in our study originated from IgM. Wetli et al. conducted immunological studies in 40 heroin addicts. Serum IgM and IgG increased by 80% and 37.5%, respectively [48]. Therefore, our study is in accordance with previous research that related heroin addiction with inflammation. Vitronectin, a glycoprotein found in the plasma and the extracellular matrix, has been reported to be a platelet specific protein, and plays a role in the formation of stable platelet aggregates [49], [50]. Previous studies have shown that the use of narcotics resulted in platelet activation [51]. Intravenously applied microcrystalline drugs (heroin) may cause microlesions of the triscupid valve endothelium [52], while endothelium dysfunction triggers platelet activation. Therefore, alterations in vitronectin abundance may reflect intravenous administration of heroin.

In summary, the 2-DE/tandem MS approach applied in the present study provided a useful tool to identify proteins related to a particular disease state, and to provide clues for in-depth analyses of proteins of interest. By use of this strategy, we were able to show, for the first time, that T4 and its carrier protein, TTR, were elevated in the serum of heroin addicts, and that the occurrence of Hp phenotype Hp0 (anhaptoglobinemia) is associated with heroin addiction. Additional studies need to be carried out to figure out how heroin dependence affects thyroid balance. Furthermore, the currently unclear causal relationship between anhaptoglobinemia and heroin addiction warrants deeper investigation.
